# A novel conceptual model and heuristic tool to strengthen understanding and capacities for health inequalities research

**DOI:** 10.1186/s12961-020-00559-z

**Published:** 2020-05-04

**Authors:** Lucinda Cash-Gibson, Matthew Harris, Germán Guerra, Joan Benach

**Affiliations:** 1 Research Group on Health Inequalities, Environment and Employment Conditions Knowledge Network (GREDS-EMCONET), Department of Political and Social Sciences, Universitat Pompeu Fabra, Mercè Rodoreda 24 Building, Campus Ciutadella UPF, Ramon Trias Fargas, 25-27, 08003 Barcelona, Catalonia Spain; 2 Johns Hopkins University - Pompeu Fabra University Public Policy Center, Barcelona, Catalonia Spain; 3 The School of Public Health, Imperial College London, London, United Kingdom; 4 National Institute of Public Health, Mexico, Av. Universidad No. 655 Colonia Santa Maria Ahuacatitlán, C.P, 62100 Cuernavaca, Morelos Mexico; 5 Transdisciplinary Research Group on Socioecological Transitions (GinTRANS2), Universidad Autónoma, Madrid, Spain

**Keywords:** Research capacity-building, research capacity strengthening, research systems, conceptual model, health equity, health inequalities

## Abstract

**Background:**

Despite increasing evidence on health inequalities over the past decades, further efforts to strengthen capacities to produce research on this topic are still urgently needed to inform effective interventions aiming to address these inequalities. To strengthen these research capacities, an initial comprehensive understanding of the health inequalities research production process is vital. However, most existing research and models are focused on understanding the relationship between health inequalities research and policy, with less focus on the health inequalities research production process itself. Existing conceptual frameworks provide valuable, yet limited, advancements on this topic; for example, they lack the capacity to comprehensively explain the health (and more specifically the health inequalities) research production process at the local level, including the potential pathways, components and determinants as well as the dynamics that might be involved. This therefore reduces their ability to be empirically tested and to provide practical guidance on how to strengthen the health inequalities research process and research capacities in different settings. Several scholars have also highlighted the need for further understanding and guidance in this area to inform effective action.

**Methods:**

Through a critical review, we developed a novel conceptual model that integrates the social determinants of health and political economy perspectives to provide a comprehensive understanding of how health inequalities research and the related research capacities are likely to be produced (or inhibited) at local level.

**Results:**

Our model represents a global hypothesis on the fundamental processes involved, and can serve as a heuristic tool to guide local level assessments of the determinants, dynamics and relations that might be relevant to better understand the health inequalities research production process and the related research capacities.

**Conclusions:**

This type of knowledge can assist researchers and decision-makers to identify any information gaps or barriers to be addressed, and establish new entry points to effectively strengthen these research capacities. This can lead to the production of a stronger evidence base, both locally and globally, which can be used to inform strategic efforts aimed at achieving health equity.

## Introduction

The social, economic and political contexts in which we live generate and maintain the social hierarchies of power and access to resources that are embedded in institutional settings and policies that create socioeconomic positions [1]; these upstream social mechanisms, or the so-called ‘structural determinants’ operate through intermediary (e.g. social, occupational) determinants that shape the distribution of risk factor exposures and social vulnerabilities in a population [1]. These processes, which are generated and maintained by “*unjust social arrangements*” [2], then become embodied by individuals, and can lead to avoidable and unfair systematic differences in health, within and between communities and countries (i.e. health inequalities (HI)) [1, 3, 4]. In this article, we use the term ‘health inequalities’ to refer to all of the following terms: HI, health disparities, health inequities and social inequalities in health.

Over the past several decades, HI have increased, along with a global awareness and evidence about this complex phenomena [5], provoking the formulation of recurring questions concerning their potential explanations – questions that all countries should answer to be able to develop effective solutions to tackle HI [6]. For example, why are there considerable inequalities in the opportunities to be healthy, between and across societies? What are the causes and conditions that lead to HI? Where and how can we intervene to improve health and well-being for all?

A prerequisite for answering these questions is investment in local capacities for HI research to be able to produce a strong evidence base to potentially inform effective policies and interventions aiming to address HI. Although the claiming of this need goes back in time, a particular emphasis was placed in the 1990 report by the Independent Commission on Health Research for Development [7], which showed major gaps in global health research and in the monitoring and evaluation of public health needs, particularly in low- and middle-income countries, and advocated for the examination of the health scientific production process itself to expand country-specific health research and its usage to improve health and health equity.

The HI research production and usage processes are important to support effective action to address HI, yet most of the current research and models focus on understanding the interplay or relationship between HI research and HI policy and action [8, 9], with less emphasis being placed on understanding how HI research itself is produced. Nevertheless, research on HI research has been growing in interest over the past few of decades, particularly in trying to establish the necessary capacities to produce HI research at the local level in different global settings [10–16]. This interest was encouraged by the final report of the WHO’s Commission on Social Determinants of Health (SDH), entitled ‘Closing a Gap in a Generation’ [17], which presented a number of recommendations to achieve health equity, including strengthening the global and local SDH and HI evidence base and research capacities.

Scientific production is considered to be a good proxy indicator of research capacity; within the HI research field itself, substantial inequalities have been found to exist between countries and world regions, in terms of the volume of production and collaborative dynamics [5]. These findings raise further questions that need to be answered; for example, why do some countries (and potentially also certain regions and institutions within countries) produce more HI research than others, particularly when HI exist everywhere? Why do some countries, despite similar level of financial resources, seem to be more ‘productive’ in this research field than others? What determines the capacity to produce HI research at the local level, in different settings? What mechanisms are involved in this process? How can local HI research capacities be strengthened? To attempt to answer these questions, the HI research production process itself needs to be better understood.

The health research systems (HRS) and policy field has been driving the thinking on how health research is produced, which is a useful starting point to try to analyse how HI research is produced. Several definitions and conceptual frameworks on national HRS and how to strengthen health research capacities have been proposed. Deciding which explanatory frameworks to use can have important implications for how one envisions the practical possibilities to proceed [18].

For example, the work by Pang et al. (on behalf of WHO) [19] was an important step forward in trying to simplify the complex systems and processes through which health research is produced to improve population health and health equity, and to establish the attributes, functions and goals of HRS, to guide the development of further operational work. Pang et al. define an HRS as “[t]*he people, institutions, and activities whose primary purpose in relation to research is to generate high-quality knowledge that can be used to promote, restore, and/or maintain the health status of populations”* ([19]. p. 816).

However, the related conceptual framework [19] presents an oversimplification of HRS, and lacks the capacity to comprehensively explain the health (and HI) research production process at the local level, thereby providing limited resources to be able to comprehensively assess these research capacities at the local level. Specifically, it fails to sufficiently account for the essential components, pathways, determinants and dynamics that are likely to be involved in creating and producing this type of research, nor does this conceptual framework consider the vital role of context and its different levels (i.e. historical, socio-cultural and eco-political choices, decisions and actions, as well as institutions within countries and regions that have shaped how HRS have emerged and developed) [1, 20].

Since Pang et al.’s [19] initial work, “*understanding local context*” has been recognised as a key component of research capacity assessments and strengthening initiatives [21]. Furthermore, a study in Guinea Bissau [22] assessed how the national HRS has developed and evolved over time, and highlights a number of important, yet often overlooked, factors that assist to provide context to the current capacity of the national HRS. For example, the authors highlight the role of history, politics and power struggles, as well as war and conflict, international development and epidemics, amongst others. Such contextual factors are likely to be highly relevant to consider when trying to understand and evaluate the current capacity to produce heath and HI research, in other post-colonial and post-conflict settings, for example.

In addition, a study in Palestine [23] found that the conceptual understanding of national HRS amongst national stakeholders varied, and was not fully aligned to the work of Pang et al. [19], concluding that clearer conceptualisation and definitions (and awareness of them) are needed to potentially improve the understanding of national HRS and facilitate progress in strengthening these research system capacities. Another study in the Eastern Mediterranean region found similar results [24].

Subsequent tools that build on the work by Pang et al. [19] have been developed [25–30], which share similar shortcomings in terms of guiding the development of further operational work. Other conceptual frameworks focused on strengthening health research capacities acknowledge that different levels of research, and research capacities, are involved in the health (and HI) research production process [3, 31–33]; however, these conceptual frameworks also present similar, limited specifications of how these research capacities are created, what factors shape or condition them, and how these different levels of research and capacities connect and interact to produce health (and HI) research at the local level, thus limiting their ability to be empirically tested in the design of integral strategies aiming to strengthen these capacities in different settings. Furthermore, a systematic review assessed the main approaches used in the health research capacity strengthening field and found insufficient insights on how sustainable national HRS are formed, limited guidance on how to address research capacity gaps and persistent ineffective strengthening strategies being utilised [34].

These challenges, both in developing comprehensive HRS analyses and effective strategies to strengthening health (and HI) research capacities, seem partially due to a limited conceptual understanding of HRS and the research production process(es). This has likely reduced the scope of knowledge necessary to make progress in strengthening these research capacities but also in developing effective multisectoral interventions to promote health equity.

The additional challenge with HI research is that HI are theoretical, empirical and practically complex [12]; therefore, to establish in-depth causal explanations, HI research often requires going beyond the use of traditional (bio)medical models of health and disease, discipline-specific theories, concepts and methods [35], and specific risks factor analyses as well as traditional hierarchies of evidence, all of which produce useful, but often ‘fragmented’ or partial, assessment of the complex problem(s) [36–38]. Instead, the development and application of integrated, transdisciplinary approaches are needed [12, 35], which include innovative methodological and theoretical approaches [12, 35, 39] and “*jointly developed*” conceptual models and frameworks that synthesise discipline-specific perspectives from the socio-political to the biological level and from the macro to the micro level [18, 35].

As such, in order to attempt to address the HI research production process knowledge gap, we present a novel conceptual model that comprises an intertwined, comprehensive approach to understand how HI research (and research capacities) are produced; by using the SDH and political economy perspectives, we build an intricate theoretical understanding of HRS, the HI research production process and research capacities at the local level. This model incorporates a number of additional aspects that have not been included in existing models/frameworks and can serve as a heuristic tool to guide HI research assessments at the local level.

Our aim is to provide the basis for new understanding and more focused empirical questions on how to strengthen the HI research production process, related research capacities and HRS in different settings, which in turn might eventually lead to breakthroughs in action towards achieving health equity.

## Materials and methods

We conducted a critical review [40] to evaluate the scientific and grey literature related to capacity-building/strengthening, HRS and HI research to develop our conceptual model. Whilst reviewing the selected literature, snow-balling search techniques were also used to identify any additional literature that may provide further critical reflections on these topics.

The public health analysis under the lens of political economy is a potent approach useful to understand HI and how people’s opportunities for health are conditioned by social, eco-political and power structures, beyond control of the individuals affected [3, 6] and can provide useful knowledge to improve the effectiveness of global public health policy analyses and action [41]. Analogously, it is also useful to understand how the opportunities and access to resources to produce HI research are conditioned in a given context. This perspective can prompt novel research questions to challenge the status quo of the distribution of resources and power in HI research structures and practices, and to explore potential ways to modify these conditions [6].

Our conceptual model describes the potential components, determinants and pathways through which HI research is created and aims to achieve a better understanding of the context within which HI research is produced at the local level; the main determinants and components of HRS and capacities for HI research; the relationship between these main determinants and components, and the production of HI research, clarifying the pathways that may lead to improvements in health equity; use of the model for evaluating local capacities for HI research; and identification of entry points for interventions aiming to strengthen capacities for HI research.

## Results

**Fig. 1:**
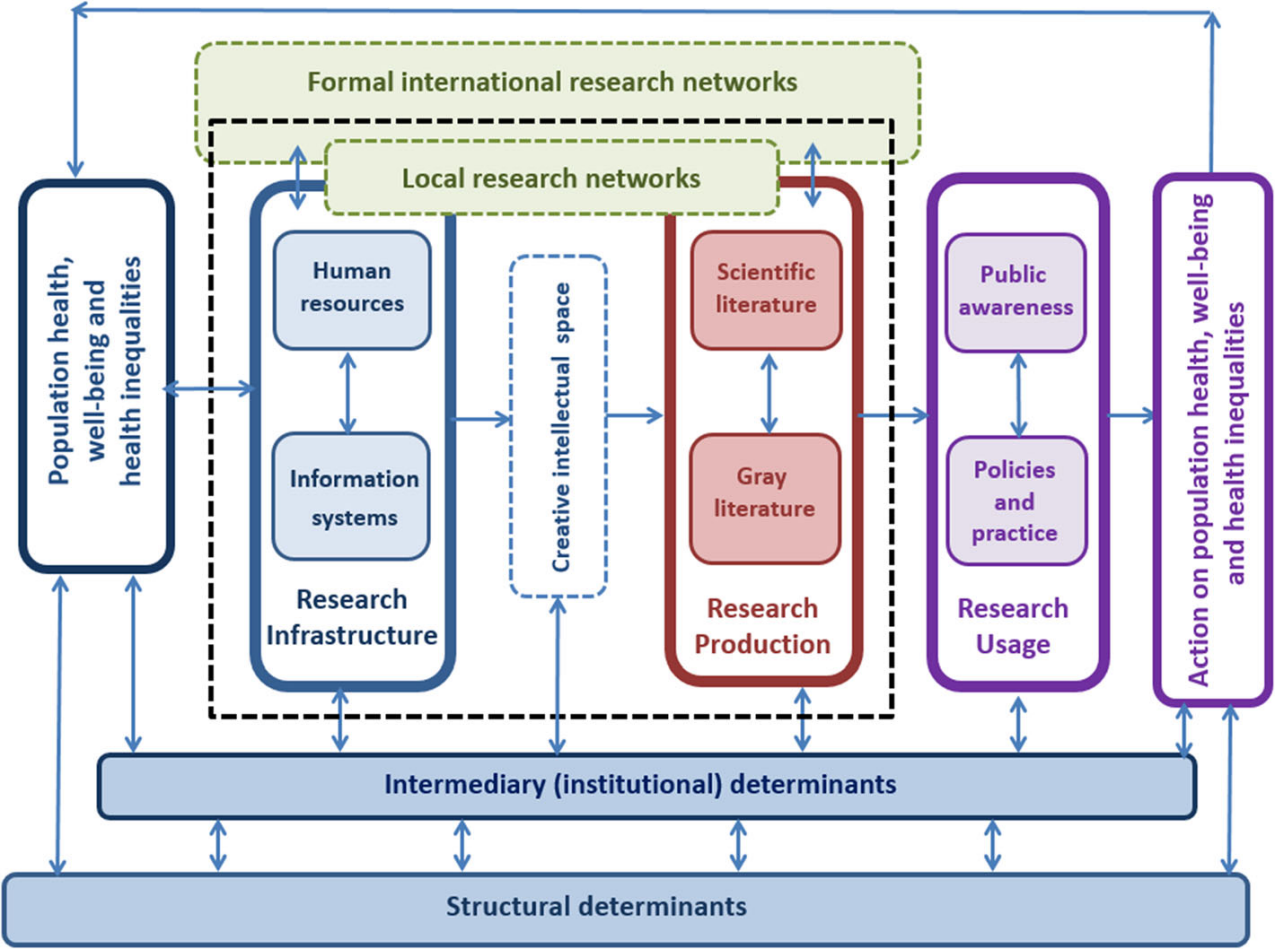
Conceptual model of a local health research system, with a focus on how health inequalities research and research capacities are produced

Figure 1 depicts our conceptual model of a local HRS along with the potential processes involved in creating and producing HI research and HI research capacities as well as how this relates to HI research usage and action; however, this latter process is not the focus of our study. Arrows indicate the pathways involved and the direction of activity.

Just as it is recognised that there are sets of structural determinants that condition people’s health opportunities [1], our conceptual model proposes that there are also (different) sets of structural determinants which operate through (different) sets of intermediary determinants to condition and shape HI research practices and HRS opportunities to produce HI research, and consequently the opportunities to address HI in a given context.

We consider the core of HRS and HI research capacity to be comprised of research infrastructure and research production (indicated by dotted lines in Fig. 1). The HI research infrastructure is composed of two multifaceted subcomponents, namely HI human resources and integrated health and socio-demographic information systems, which can come together within a creative intellectual space and interact (as appropriate, depending on the research questions proposed), to enable critical HI research to be created and produced.

Furthermore, we consider financing, stewardship and governance to be threads that run throughout the HRS, each with their own internal dynamics that will shape the opportunities and access to the resources available, and which can enable or disable the HI research production process at various points.

To explain our model, we start from HI research production (i.e. the outcome of interest in this study) and elaborate backwards to cover the main pathway(s), components and determinants as well as the dynamics potentially involved in this overall process.

### Research production

As mentioned, an indication of research capacity is the counting of scientific international peer-review journal publications [30]. Whilst grey literature (e.g. briefs, reports) can also be produced, disseminated and used alongside scientific (HI) research, it is a separate process and not the focus of our paper.

### Creative intellectual space

To create critical HI scientific research, the intellectual and creative autonomy of HI researchers needs to be fostered through a supportive research infrastructure at the systemic and institutional (macro and meso) levels. This includes career pathways and (transdisciplinary) research training so that HI researchers are given the opportunity and resources, including sufficient time [8], to reflect and pose relevant innovative questions, pursue critical HI research on complex global-societal issues, and be in a better position to be able to explore potential ways to modify these inequitable conditions and outcomes. Such issues include HI and/or the (unequal) distribution of resources and power in social structures, between and within countries, which are maintained by contemporary global and societal norms and policies, and ‘privileged’ actors [42] as well as the micro level power struggles that manifest and impact people’s health and well-being [6].

However, several scholars have mentioned the “*limited academic freedom*” there is in the health and social science research fields in certain countries due to academic institutional structures, ideologies and dependence for research funding, and the impact this can have on the framing of the HI research design and findings [43, 44].

Critical HI research requires the application of integrated transdisciplinary approaches, such as an eco-social lens [45], to consider the social, historical and ideological forces and power structures that can maintain and (re)produce HI. Additionally, it requires going beyond the privileging of scientific knowledge created in certain (often higher-income country) settings [46, 47] and the adoption of privileged, hegemonic methodological (and philosophical) approaches often used in public health and HI research due to “*their perceived strength in establishing cause and effect*” ([36], p. 252–253), which provide only partial accounts of social reality, or of a complex social phenomenon [38], resulting in a limited contribution to the knowledge required to address HI in a given setting, globally [31].

At the global scale, efforts aiming to strengthen HI research capacity need to consider these dynamics so as to determine how to develop enabling HI research conditions and individual research skillsets as well as how to overcome the “*epistemic injustices*” and deep-seeded “*unconscious biases*” still prevalent in varying degrees in global health research production and research practices [46, 48–51] and in particular the HI research field [5, 38].

### Research infrastructure

As existing research highlights, development of HI research infrastructure requires a conducive research environment and the provision of resources such as facilities, financial research support, and scientific leadership as well as enabling career structures, good research management, and access to technical information and equipment [33, 52], amongst other things. A key component of HI research infrastructure is a critical mass of skilled workforce that, through adequate (ideally transdisciplinary) training, mentoring and research infrastructural support [53], will have the competences to understand and assess the broader determinants of HI, to design, lead and conduct critical HI research, as well as to establish sustainable research institutions, teams, and networks, and co-develop effective solutions to address HI at local level [33]. These have been identified as pending needs in many countries and regions around the world [12, 31, 54].

In addition, the capacity and governance to consistently collect, manage and report data at the macro, meso and micro level, across time, are also pending issues in many countries [5, 55, 56]. However, with the limited data that is currently available, the WHO Global Observatory on Health Research and Development calculates that, on average, higher-income countries have 73 times more health researchers than low-income countries [57]. This highlights the average size of the human resource capacity gap that is likely to exist between certain groups of countries, globally, in being able to undertake health and HI research. Furthermore, substantial gaps in data and human resource capacity are also likely to exist within countries, which have not been reported.

At the same time, countries and local regions need to be able to describe and measure the extent of HI and their determinants, understand and monitor their evolution overtime, and use this evidence to design and adjust interventions to maximise the health benefits for all [17, 33, 58]. This requires reliable, disaggregated and integrated health and socio-demographic data, information systems and routine monitoring mechanisms, supported by human resources. Such information systems can assist researchers and decision-makers to identify entry points for HI intervention, evaluate the impact of policies and prioritise the use of resources to work towards health equity [59].

Global efforts have been made to enhance the equity orientation of national health information systems and to build HI observatories, which have also identified several pending challenges to be addressed and which can provide useful learning for other settings [54, 58–63]. For example, an evaluation of the capacity of Mozambique’s national health information systems to monitor and measure health equity [64] identified significant gaps in the availability of disaggregated equity stratifiers to be able to measure and monitor the targets for United Nation’s Sustainable Development Goal three, which is focused on ensuring healthy lives and promoting well-being [65, 66]. Such technical gaps, which are likely to exist in similar low-income country settings, not only inhibit the monitoring and measuring of HI, health equity and other related outcomes themselves, but also the potential design and adjustment of much needed multi-sectoral policy changes in these settings [64, 66].

### Intermediary determinants of HI research

Local research agendas and prioritises are not always aligned with, and driven by, local population health and well-being needs. Institutions also play a key role in the politics (understood as the exercise of power between groups) of health [67], in the process of HI (re)production, and in the HI research production process itself, acting as ‘vectors of power’ that is exercised and controlled by hegemonic groups [44, 68]. Research funding institutions, for example, do not simply provide and allocate research funding resources, they also play a role in framing and steering research agendas and priorities [68], and in deciding what type of research gets supported (or not), where and by whom, as well as the ‘appropriateness’ of the research frame used, often in line with certain ideologies [32, 33, 47, 52, 69, 70]. By ideology, we mean a system of value judgments and beliefs that shape how research, and policy, is conventionally developed and carried out [44, 71].

Furthermore, scholars have pointed out that (research) institutions at all different levels, including universities, can be deeply ideological, which can sometimes (negatively) impact the HI scientific discourse, and researcher academic careers in the case of those interested in potentially controversial topics such as HI [43, 44].

Applying a political economy perspective and an integrated transdisciplinary approach to the (HI) knowledge production process, for example, allows one to see that it is not a value-neutral, apolitical and purely scientific process [43, 44], rather it is shaped by “...*ideological values, political and power relations, and economic forces*” ([44], p. 916). However, so far, these types of approaches, reflections and considerations have been limitedly applied to HI research [44, 68], and even less so to the HI research production process and HI research capacities.

### Structural determinants of HI research

It is understood that structural determinants of HI exist within specific political and historical contexts, which consist of a number of interacting macro-level factors or determinants (e.g. macroeconomic and public policies, socio-cultural values and epidemiological conditions, among others) that change over time, and can generate, configure and maintain social structures, and exert influence (and power) at an intermediary (meso) level(s); this, in turn, can be embodied and can condition the subsequent opportunities to produce certain (health) outcomes at the individual (micro) level [1].

Our conceptual model therefore proposes that there are also sets of structural determinants that operate through intermediary determinants to condition and shape domestic HI research practices, and HRS opportunities to produce HI research, which consequently condition and shape the opportunities and access to resources to be able to potentially address HI in a given context.

Additionally, within and across social contexts, the views, values and ideologies around HI differ [1, 44], which, as mentioned, likely impacts the type and degree of action taken to address them [16, 26, 33]. For example, HI are either seen as ‘natural’ and ‘inevitable’ outcomes of individual (lifestyle) choices and genetic differences, where the State has less ‘responsibility’ in creating the necessary changes [72–74] or as a social injustice that needs to be tackled by all at various social levels [72]. The first perspective is thought to be partly due to the fact that the public health field has traditionally been dominated by professionals trained (only) in medicine or biology, and who focus on the “*biomedical models of heath and disease*” (rather than the ‘social models’), where health is considered as the absence of disease (and/or a commodity), and the distribution of (‘poor’) health and HI are predominantly the result of (‘poor’) individual choices and behaviours [72]. The biomedical models do not acknowledge the role of upstream structures and (class, gender and race/ethnicity) power relations within which individual agency exists and can be shaped by [1, 43, 72, 73]. As a result, “*socio-structural violence*” is often committed ([74], p. 239), where the victims of HI are often blamed and stigmatised for their own injuries [43, 74] and political attention and interventions are mainly directed downstream towards promoting (and correcting ‘poor’) individual lifestyle choices and behaviours, and improving healthcare services [72] – despite the health system being just one of the many intermediary determinants of HI [1]; this has occurred in the United Kingdom, for example, during various historical periods [74–76].

Underpinning all of this are not only divergent views of what action is possible, but also different institutional and individual ideologies and values about what is considered to be socio-politically desirable in society (i.e. egalitarian versus individualism) [44], including giving more or less importance to issues related to territory, class, gender, ethnicity, etc. As such, it becomes clear that the way HI are considered, and the subsequent action taken to address them, is highly political [72].

Furthermore, under globalised neoliberalism [77], changes in the roles and regulations of the state, foreign affairs and the market have led to the increasing influence of global eco-political conditions in domestic decisions and global governance issues, i.e. global political determinants, that impact on health and HI [6, 78]. These constitute important dimensions of ‘context’ that need to be analysed and considered, alongside the strategies pursued by actors and institutions involved in such global and local arrangements [1].

### Research networks

Our model presents how different types of (local and international, formal and informal) research networks can interact with HRS to pool and mobilise differential individual and institutional resources and capacities to strengthen research capacities [79]. These networks bring new conditions, pathways and relations to the HI research production process as well as new individual behaviours, interests and micro (power) struggles to the research process [8, 76].

Formal, international research networks, for example, can pool and mobilise international and local resources and capacities, and have become important players in strengthening research capacities for research, particularly in low- and middle-income countries. Examples of such types of networks include vertical research projects, centres of excellence, and global North–South (and more recently, global South–South) research partnerships and consortia [34].

## Discussion

Firstly, we find that the distinction must be made between the processes of producing, and of using, health or HI research. For example, Pang et al. [19] consider both the ‘research production’ process and ‘research usage’ process as the two main processes and goals of HRS, but then consider ‘producing and using research’ to be one single HRS function. However, this perspective takes a linear view on how health or HI research is produced, disseminated and incorporated into policy and practice to improve health and well-being [8, 80].

In reality, as many political and social scientists discuss [8, 81], the process of ‘research usage’ is neither linear, nor simultaneous, but rather influenced by a number of other factors and stakeholders that are intertwined with institutional and individual ideologies, values and interests [8, 76, 82]. Therefore, instead, we propose that the main goal and function of HRS are to produce health or HI research that may or may not be used, and we conceive ‘research usage’ to be a separate process and secondary goal (and function) of HRS, which is beyond the scope of our paper.

Secondly, HI and health equity are inseparable from power and politics [72], which means that action on HI, including creating and producing HI research, is a political process [26, 83]. Yet, the political determinants of health and HI have been largely neglected and marginalised from mainstream public health debate and analyses [31, 34, 72, 84], this includes an absence of questions related to politics and power dynamics within and between societies and countries [44]. As one study in Ethiopia highlights [85], if and when politics is referred to in mainstream public health research, it is often in regard to whether there is political commitment or not, rather than going deeper into the political context to consider how politics impacts health, HRS and the related research practices, or how internal power relations could be changed to achieve better health (and related research) outcomes [85, 86]. This is thought to be due, in part, to what we mentioned previously about the two main models of health, disease and HI. However, between these two main stances, there are also more nuanced perspectives. For example, some may consider the topic to be too complex (i.e. a “*wicked*” problem) or “*too political*” ([73], p. 115), not covered within their “*disciplinary skill set*” and/or not in their “*own interest*” to question or challenge HI, the status quo or their positionality [43, 73].

Scholarly debates over HI research findings, ultimately epidemiological and ontological debates over “*causality and causal relationship*” [35, 38] and the relative importance of individual behaviour and action and social structures [3], have stated that these issues are not only of scientific interest, but can also be used to push for certain policy responses and, therefore, hold significant political implications [35, 87]. For example, this has been highlighted in the case of the United Kingdom over various historical and political periods [8, 75, 81, 87].

Thirdly, formal international health research networks are also shaped and conditioned by underlying historical and contemporary geo-political power relations that exist amongst country partners at institutional and individual level [47, 70, 88]. These types of networks are often led by external partners (linked to funding sources) [20, 28, 42, 47] and are considered to be valid approaches to enhance local HI research capacities, with potentially mutual benefits for all involved, providing that certain ethical principles are followed and contractually established [12, 89, 90].

However, there are also concerns that, as unanticipated consequences, these new research environments can potentially create research dependence, “*intellectual colonialism*” [70, 91, 92] and/or establish parallel structures that bypass domestic research systems [15, 22], which can restrict and/or erode local sovereignty [16] and exasperate the very problems they claim to aim to solve [33, 93]. Yet, concepts of power (and power struggles) at the meso- and micro-levels within these networks are insufficiently recognised, and need to be addressed to be able to determine to what extent countries, institutions and researchers have the power, capacity (including equitable access to opportunities and resources) and agency to determine if, and what type of, HI research is produced at local level. This need to acknowledge and address unequal power relations in public health research collaborations has been highlighted in a study conducted in Zambia [94], and in a systematic review on managing (formal, international) health research capacity strengthening networks [95].

Fourthly, applying a political economy perspective to public health analyses can help to assess the distribution of power and resources within HI research and its development [6], despite public health researchers and practitioners not being typically trained to conduct this type of analysis [96]. A political economy perspective has been discussed and advocated for in the context of the Sustainable Development Goals and the ‘leaving no one behind’ agenda [65, 96], which acknowledges the need to challenge the “*enormous disparities of opportunity, wealth and power*” that exist globally [65, 96]. This also requires integrated, interdisciplinary and intersectoral collaborations and approaches to understand and inform programmatic action on the various commercial, political, economic, environmental and social determinants of HI [66].

Fifthly, in addition to identifying the components, determinants and pathways involved in this process, the identification of mechanisms and causal linkages that are triggered in certain contexts, and which can lead to the outcome of interest [97] (i.e. stronger HI research capacities and increased HI research production in this case) is also crucial. This type of in-depth understanding about causal explanations can be used to inform the strategic development of more effective strategies to strengthen this research process and its related capacities. This is important since research is more than just a tool to generate new knowledge, it can also serve as a strategy to advance population health and social change [44].

As such, scholars have argued for more HI research to go beyond what can be “*observed or measured*” via positivistic quantitative approaches or “*perceived*” by study participants via interpretivisitic qualitative approaches which only provide descriptions and partial understanding of social reality [38]. Broader epistemology and ontological approaches, such as realist approaches, are thought to be useful since they try to consider both structure and agency [38, 98], and to reconcile the tension between scientific objectivity (which promotes neutrality or value-free science) and value judgments [44], amongst other things, to establish more in-depth causal explanations and understanding of the complex issue under study [38]. Realist approaches have started to be used to evaluate complex health and social issues and interventions [36, 38, 97]; such approaches should also be considered by researchers and decisions-makers in combination with our guiding conceptual model when planning local HI research capacity assessments and evaluations.

Lastly, critical reviews are useful to develop a hypothesis or model that acts as a starting point for further evaluation with the aim of critically evaluating the potential value from the aggregate literature to provide a new phase of conceptual development and subsequent testing. Whilst these critical interpretations are essentially subjective, emphasis is placed on the conceptual contribution of each item of the included literature [40], serving as a value method for our article.

To conclude, our model was purposefully designed to understand the HI research production process at a global level to ensure its relevance for different settings since HI research capacity challenges exist globally [5, 63]. An application of our model to a specific country or local setting would require an exhaustively defined context-based model that exceeds the limits of this paper. However, it is expected that such application should be developed in the future to empirically test and analyse our model, and guide further in-depth analyses of the HI research production process in different contexts. For example, we encourage the development of in-depth case study analyses, using realist approaches, to identify key contextual factors and mechanisms involved in creating and producing HI research and research capacities. This knowledge can support more pragmatic thinking on what type of intervention could effectively strengthen the HI research production process and HI research capacities, where, how and for whom.

## Conclusion

Despite increase evidence on HI over the past decades, efforts are still urgently needed to strengthen capacities to produce HI diagnoses, and to establish entry points for interventions aiming to address HI and population health needs. Comprehensive conceptual understanding of the HI research production process is a vital first step, yet current research and models have mainly focused on the HI research utilisation process rather than on the HI research production process itself.

A number of existing conceptual frameworks, focused on understanding how health research is produced, used and strengthened, provide valuable yet limited advancements in this area. For example, they lack the capacity to comprehensively explain the potential pathways, components, key determinants and dynamics involved in the health, and more specifically the HI, research production process at the local level, thus limiting their ability to be empirically tested and to provide practical guidance on how to strengthen the HI research production process and related research capacities in different settings. Several scholars have also identified insufficient insights in these areas and have highlighted the need for further understanding and guidance in this broad topic.

To fill this knowledge gap, we developed a novel conceptual model that integrates the SDH and political economy perspectives to provide a comprehensive understanding on how HI research is potentially produced (or inhibited) at the local level. Our model represents a global hypothesis on the fundamental processes, and key components, determinants and dynamics involved, and can serve as a heuristic tool to guide the assessment of the HI research production process and research capacity at the local level. The application of this model could assist to identify information gaps and barriers, and provide the basis for new understanding and more focused empirical questions on how to strengthen HI research capacities.

We encourage researchers and decision-makers working in this broad area to test and adapt our model to different local contexts, potentially in combination with a realist approach, to develop more comprehensive assessments of local capacities for HI research as well as to establish the potential mechanisms and causal linkages involved. Such information might assist in establishing new entry points to strengthen HI research capacities and the evidence base, which in turn can be used to inform more locally relevant interventions aiming to address HI as well as to inspire the praxis and social transformation necessary to achieve health equity.

## Data Availability

Not applicable.

## Abbreviations

HI: health inequalities
HRS: health research system
SDH: social determinants of health
